# 2-[(2-Hydr­oxy-2,2-diphenyl­ethyl)(meth­yl)amino]-*N*,*N*-dimethyl­ethanaminium bromide

**DOI:** 10.1107/S1600536809002414

**Published:** 2009-01-23

**Authors:** Viktoria H. Gessner, Christian Däschlein, Carsten Strohmann

**Affiliations:** a Anorganische Chemie, Technische Universität Dortmund, Otto-Hahn-Strasse 6, 44227 Dortmund, Germany

## Abstract

The title compound, C_19_H_27_N_2_O^+^·Br^−^, is the hydro­bromide of the trapping product of lithia­ted *N*,*N*,*N*′,*N*′-tetramethylethylenediamine (TMEDA) with benzophenone. Thereby, the N atom of the NMe_2_ group is selectively protonated and the respective trapping product represents a potential tridentate ligand with one O and two N donor atoms. The H atoms at N (H2N) and O (H1O) are involved in hydrogen bonds with the Br^−^. The mol­ecular structure shows all donor atoms to be arranged on one side of the mol­ecule, thus indicating a potential threefold coordination of a Lewis acid.

## Related literature

For related literature on direct deprotonation of tertiary amines, see: Strohmann & Gessner (2007*a* ▶,*b* ▶,*c* ▶, 2008*a* ▶,*b* ▶), Gessner & Strohmann (2008 ▶); Bojer *et al.* (2007 ▶); Karsch (1996 ▶); Strohmann *et al.* (2008 ▶); Köhler *et al.* (1987 ▶); Arnold *et al.* (2002 ▶).
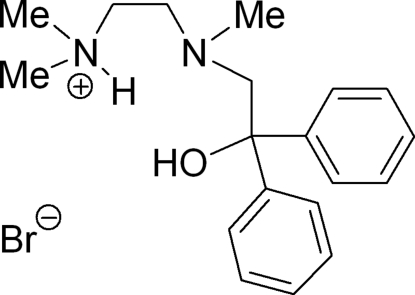


## Experimental

### 

#### Crystal data

C_19_H_27_N_2_O^+^·Br^−^
*M* *_r_* = 379.34
Orthorhombic, 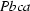

*a* = 7.119 (2) Å
*b* = 15.515 (3) Å
*c* = 33.585 (7) Å
*V* = 3710 (1) Å^3^
*Z* = 8
Mo *K*α radiation
μ = 2.22 mm^−1^
*T* = 173 (2) K
0.4 × 0.2 × 0.2 mm

#### Data collection

Bruker APEX CCD diffractometer
Absorption correction: empirical (using intensity measurements) (*SADABS*; Bruker, 1999 ▶) *T*_min_ = 0.440, *T*_max_ = 0.635
78737 measured reflections
3640 independent reflections
3200 reflections with *I* > 2σ(*I*)
*R*_int_ = 0.064

#### Refinement

*R*[*F* ^2^ > 2σ(*F* ^2^)] = 0.037
*wR*(*F* ^2^) = 0.099
*S* = 1.07
3640 reflections
219 parameters
H atoms treated by a mixture of independent and constrained refinement
Δρ_max_ = 0.49 e Å^−3^
Δρ_min_ = −0.43 e Å^−3^

### 

Data collection: *SMART* (Bruker, 2001 ▶); cell refinement: *SAINT-Plus* (Bruker, 1999 ▶); data reduction: *SAINT-Plus*; program(s) used to solve structure: *SHELXS90* (Sheldrick, 2008 ▶); program(s) used to refine structure: *SHELXL97* (Sheldrick, 2008 ▶); molecular graphics: *ORTEP-3* (Farrugia, 1997 ▶); software used to prepare material for publication: *SHELXL97*.

## Supplementary Material

Crystal structure: contains datablocks I, global. DOI: 10.1107/S1600536809002414/im2095sup1.cif

Structure factors: contains datablocks I. DOI: 10.1107/S1600536809002414/im2095Isup2.hkl

Additional supplementary materials:  crystallographic information; 3D view; checkCIF report

## Figures and Tables

**Table 1:** Hydrogen-bond geometry (Å, °)

*D*—H⋯*A*	*D*—H	H⋯*A*	*D*⋯*A*	*D*—H⋯*A*
N2—H2N⋯Br	0.87 (4)	2.52 (4)	3.276 (3)	145 (4)
O—H1O⋯Br	0.69 (3)	2.76 (3)	3.398 (2)	156 (3)

**Fig. 1.**
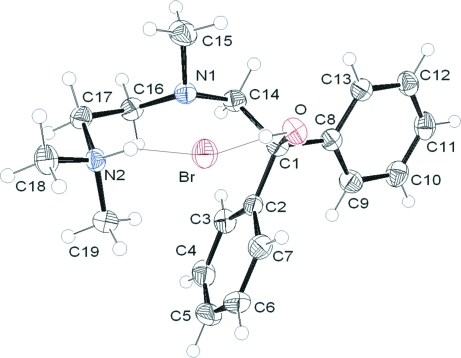
ORTEP plot of 2-[(2'-Hydroxy-2',2'-diphenylethyl)-N'-methyl(amino)]-N,N-dimethylethanaminium bromide. Thermal ellipsoids are drawn at the 50% probability level.

## supplementary crystallographic information

### Comment

Due to their application in many fields of chemistry, the preparation of nitrogen ligands is still of great interest in synthetic chemistry. Recent investigations have proved the direct deprotonation of methylamines to be a synthetically very useful method for functionalizations and thus for the synthesis of nitrogen ligands. Further donor atoms as well as further stereocenters can easily be introduced to the molecule. Only few tertiary methylamines are known for their reactivity towards such a direct lithiation reaction, amongst (*R*,*R*)-TMCDA (Strohmann & Gessner, 2007*a*,*c*, 2008*a*; Strohmann *et al.*, 2008), PMDTA (Strohmann & Gessner, 2007*b*) and TMEDA (Gessner & Strohmann, 2008; Köhler *et al.*, 1987). Generally, this type of reaction is an undesired side reaction resulting in the loss of base and leading to side products, *e.g.* in deprotonation or addition reactions with lithiumalkyls (Arnold *et al.*, 2002). However, this decomposition of the Lewis base can also be used synthetically for the preparation of new ligand systems (Karsch, 1996; Bojer *et al.*, 2007; Strohmann & Gessner, 2008*b*).

The title compound 2-[(2'-Hydroxy-2',2'-diphenylethyl)-*N*'-methyl(amino)]-*N*,*N*-dimethylethanaminium bromide can be synthesized by direct deprotonation of TMEDA with *tert*-butyllithium and the subsequent trapping reaction with benzophenone. The yield of this lithiation reaction is limited due to the competitive deprotonation of the ethylene bridge of the ligand. Treatment of the resulting amino alcohol with lithiumbromide under acidic conditions gives the hydrobromide as colorless crystals. The structure indicates a potential threefold coordination as all donor atoms (two N atoms and one oxygen) are arranged at the same side of the molecule.

2-[(2'-Hydroxy-2',2'-diphenylethyl)-*N*'-methyl(amino)]-*N*,*N*-dimethylethanaminium bromide crystallizes in the othorhombic crystal system, space group Pbca. The asymmetric unit contains only one molecule of the monomeric compound.

### Experimental

2-(2-Dimethylaminoethyl)(methyl)amino-1,1-diphenylethanol and an equivalent amount of LiBr were dissolved in a mixture of acetone and a few trops acetic acid and stored at room temperature for 24 h. After evaporation of the solvent a crystalline solid remained, suitable for X-ray studies.

^1^H NMR (400.1 MHz, D_2_O): δ = 2.17 (s, 3H; N(CH_3_)CH_2_), 2.42 (s, 6H; N(CH_3_)_2_), 2,68 (t, ^3^*J*_HH_ = 6.16 Hz, 3H; CH_2_N), 2.95 (t, ^3^*J*_HH_ = 6.14 Hz, 2H; CH_2_N), 3.43 (s, 2H; NCH_2_C(OH)Ph_2_), 6.60–7.10 (br, 2H; NH and OH), 7.20–7.24 (m, 2H; H_para_), 7.24–2.33 (m, 4H; H_arom_), 7.41–2.43 (m, 4H; H_arom_).

{^1^H}^13^C NMR (100.6 MHz, D_2_O): δ = 42.4 (HN(CH_3_)_2_), 43.7 (N(CH_3_)CH_2_), 52.9 (CH_2_N(H)(CH_3_)_2_), 55.1 (CH_2_CH_2_N(CH_3_)CH_2_), 66.6 (NCH_2_C(OH)Ph_2_), 78.1 (CPh_2_), 125.9 (C_meta_), 1276.4 (C_para_), 128.6 (C_ortho_), 145.3 (C_ipso_).

### Refinement

Refinement was accomplished by full-matrix least-squares methods (based on F_o_^2^, *SHELXL97*); anisotropic thermal parameters for all non-H atoms in the final cycles; the H atoms were calculated into idealized positions and refined using a riding model with *U*_iso_(H) = 1.2*U*_eq_(C) for CH and CH_2_ groups and 1.5*U*_eq_(C) for methyl groups. The positions of H2N and H1O were determined from the difference Fourier map and they were refined without constraints.

### Crystal data

**Table:**

C_19_H_27_N_2_O^+^·Br^−^	*F*(000) = 1584
*M**_r_* = 379.34	*D*_x_ = 1.358 Mg m^−^^3^
Orthorhombic, *P**b**c**a*	Mo *K*α radiation, λ = 0.71073 Å
Hall symbol: -P 2ac 2ab	θ = 1.2–26°
*a* = 7.119 (2) Å	µ = 2.22 mm^−^^1^
*b* = 15.515 (3) Å	*T* = 173 K
*c* = 33.585 (7) Å	Needle, colourless
*V* = 3710 (1) Å^3^	0.4 × 0.2 × 0.2 mm
*Z* = 8	

### Data collection

**Table:**

Bruker APEX CCD diffractometer	3640 independent reflections
Radiation source: fine-focus sealed tube	3200 reflections with *I* > 2σ(*I*)
graphite	*R*_int_ = 0.064
ω scans	θ_max_ = 26°, θ_min_ = 1.2°
Absorption correction: empirical (using intensity measurements) (*SADABS*; Bruker, 1999)	*h* = −8→8
*T*_min_ = 0.440, *T*_max_ = 0.635	*k* = −19→19
78737 measured reflections	*l* = −41→41

### Refinement

**Table:**

Refinement on *F*^2^	Primary atom site location: structure-invariant direct methods
Least-squares matrix: full	Secondary atom site location: difference Fourier map
*R*[*F*^2^ > 2σ(*F*^2^)] = 0.037	Hydrogen site location: inferred from neighbouring sites
*wR*(*F*^2^) = 0.099	H atoms treated by a mixture of independent and constrained refinement
*S* = 1.07	*w* = 1/[σ^2^(*F*_o_^2^) + (0.0504*P*)^2^ + 4.0375*P*] where *P* = (*F*_o_^2^ + 2*F*_c_^2^)/3
3640 reflections	(Δ/σ)_max_ = 0.003
219 parameters	Δρ_max_ = 0.49 e Å^−^^3^
0 restraints	Δρ_min_ = −0.43 e Å^−^^3^

### Special details

**Table:**

Geometry. All e.s.d.'s (except the e.s.d. in the dihedral angle between two l.s. planes) are estimated using the full covariance matrix. The cell e.s.d.'s are taken into account individually in the estimation of e.s.d.'s in distances, angles and torsion angles; correlations between e.s.d.'s in cell parameters are only used when they are defined by crystal symmetry. An approximate (isotropic) treatment of cell e.s.d.'s is used for estimating e.s.d.'s involving l.s. planes.
Refinement. Refinement of *F*^2^ against ALL reflections. The weighted *R*-factor *wR* and goodness of fit *S* are based on *F*^2^, conventional *R*-factors *R* are based on *F*, with *F* set to zero for negative *F*^2^. The threshold expression of *F*^2^ > σ(*F*^2^) is used only for calculating *R*-factors(gt) *etc*. and is not relevant to the choice of reflections for refinement. *R*-factors based on *F*^2^ are statistically about twice as large as those based on *F*, and *R*- factors based on ALL data will be even larger.

### Fractional atomic coordinates and isotropic or equivalent isotropic displacement parameters (Å^2^)

**Table:**

	*x*	*y*	*z*	*U*_iso_*/*U*_eq_	
Br	0.55743 (4)	0.697337 (19)	0.564819 (9)	0.03232 (11)	
C1	0.5689 (4)	0.93459 (17)	0.63574 (8)	0.0250 (6)	
C2	0.6972 (4)	0.88169 (17)	0.66284 (8)	0.0250 (6)	
C3	0.8583 (4)	0.9173 (2)	0.67970 (9)	0.0320 (7)	
H3	0.8892	0.9757	0.6743	0.038*	
C4	0.9747 (5)	0.8691 (2)	0.70430 (9)	0.0406 (8)	
H4	1.0839	0.8946	0.7155	0.049*	
C5	0.9321 (5)	0.7848 (2)	0.71242 (10)	0.0450 (9)	
H5	1.0122	0.7516	0.7291	0.054*	
C6	0.7726 (5)	0.7483 (2)	0.69634 (10)	0.0429 (8)	
H6	0.7427	0.6900	0.7021	0.052*	
C7	0.6550 (5)	0.79621 (19)	0.67180 (9)	0.0332 (7)	
H7	0.5451	0.7704	0.6611	0.040*	
C8	0.4435 (4)	0.99595 (17)	0.65971 (8)	0.0263 (6)	
C9	0.4565 (4)	1.0055 (2)	0.70069 (9)	0.0335 (7)	
H9	0.5476	0.9736	0.7151	0.040*	
C10	0.3373 (5)	1.0615 (2)	0.72079 (9)	0.0380 (7)	
H10	0.3473	1.0671	0.7489	0.046*	
C11	0.2045 (5)	1.1091 (2)	0.70046 (10)	0.0372 (7)	
H11	0.1250	1.1480	0.7143	0.045*	
C12	0.1884 (4)	1.09930 (19)	0.65969 (9)	0.0337 (7)	
H12	0.0965	1.1312	0.6454	0.040*	
C13	0.3059 (4)	1.04315 (18)	0.63960 (9)	0.0290 (6)	
H13	0.2928	1.0366	0.6116	0.035*	
C14	0.6829 (4)	0.98676 (17)	0.60473 (8)	0.0285 (6)	
H14A	0.5987	1.0285	0.5914	0.034*	
H14B	0.7824	1.0198	0.6186	0.034*	
C15	0.6945 (5)	0.9484 (2)	0.53493 (9)	0.0402 (8)	
H15A	0.7315	1.0066	0.5267	0.060*	
H15B	0.5572	0.9443	0.5355	0.060*	
H15C	0.7445	0.9062	0.5160	0.060*	
C16	0.9753 (4)	0.93415 (19)	0.57482 (9)	0.0289 (6)	
H16A	1.0205	0.9333	0.6027	0.035*	
H16B	1.0168	0.9890	0.5626	0.035*	
C17	1.0617 (4)	0.85949 (18)	0.55238 (9)	0.0288 (6)	
H17A	1.0360	0.8664	0.5236	0.035*	
H17B	1.1995	0.8603	0.5562	0.035*	
C18	1.0397 (5)	0.70427 (19)	0.53833 (10)	0.0345 (7)	
H18A	1.1761	0.6960	0.5392	0.052*	
H18B	1.0018	0.7198	0.5112	0.052*	
H18C	0.9767	0.6508	0.5462	0.052*	
C19	1.0432 (4)	0.7534 (2)	0.60756 (9)	0.0344 (7)	
H19A	0.9805	0.7002	0.6161	0.052*	
H19B	1.0071	0.8007	0.6253	0.052*	
H19C	1.1796	0.7453	0.6086	0.052*	
N1	0.7695 (3)	0.93059 (15)	0.57460 (7)	0.0262 (5)	
N2	0.9858 (4)	0.77440 (16)	0.56609 (7)	0.0260 (5)	
H2N	0.864 (6)	0.779 (2)	0.5669 (10)	0.044 (10)*	
O	0.4411 (3)	0.88041 (14)	0.61458 (7)	0.0298 (5)	
H1O	0.494 (5)	0.849 (2)	0.6050 (10)	0.024 (10)*	

### Atomic displacement parameters (Å^2^)

**Table:**

	*U*^11^	*U*^22^	*U*^33^	*U*^12^	*U*^13^	*U*^23^
Br	0.02792 (17)	0.03281 (18)	0.03624 (18)	−0.00315 (12)	0.00142 (12)	−0.00227 (12)
C1	0.0251 (14)	0.0217 (13)	0.0283 (14)	−0.0032 (11)	−0.0027 (12)	−0.0014 (11)
C2	0.0242 (14)	0.0256 (14)	0.0252 (13)	0.0005 (11)	0.0033 (11)	0.0001 (11)
C3	0.0301 (16)	0.0327 (15)	0.0333 (16)	−0.0008 (13)	−0.0003 (13)	−0.0044 (12)
C4	0.0297 (17)	0.062 (2)	0.0301 (16)	0.0028 (16)	−0.0057 (13)	−0.0061 (15)
C5	0.047 (2)	0.058 (2)	0.0308 (17)	0.0190 (17)	−0.0028 (15)	0.0079 (15)
C6	0.048 (2)	0.0371 (17)	0.0432 (18)	0.0083 (16)	0.0076 (16)	0.0158 (15)
C7	0.0315 (16)	0.0310 (15)	0.0371 (16)	−0.0041 (13)	0.0020 (14)	0.0034 (13)
C8	0.0249 (14)	0.0217 (13)	0.0324 (15)	−0.0050 (11)	−0.0008 (12)	−0.0002 (11)
C9	0.0331 (17)	0.0347 (16)	0.0327 (16)	−0.0002 (14)	−0.0033 (13)	−0.0013 (13)
C10	0.0387 (18)	0.0446 (18)	0.0309 (16)	−0.0019 (15)	0.0036 (14)	−0.0070 (14)
C11	0.0328 (17)	0.0306 (15)	0.0481 (19)	−0.0004 (14)	0.0075 (14)	−0.0079 (14)
C12	0.0261 (15)	0.0286 (15)	0.0464 (18)	−0.0006 (13)	0.0023 (13)	0.0022 (13)
C13	0.0281 (15)	0.0266 (14)	0.0322 (15)	−0.0029 (12)	−0.0012 (12)	0.0007 (12)
C14	0.0335 (16)	0.0203 (13)	0.0316 (15)	0.0005 (12)	−0.0005 (13)	0.0006 (11)
C15	0.0403 (18)	0.0470 (19)	0.0334 (16)	0.0054 (16)	−0.0093 (14)	−0.0049 (14)
C16	0.0276 (15)	0.0301 (15)	0.0289 (14)	−0.0067 (12)	−0.0014 (12)	−0.0005 (12)
C17	0.0252 (15)	0.0317 (15)	0.0295 (14)	−0.0032 (12)	0.0022 (12)	0.0041 (12)
C18	0.0328 (17)	0.0323 (16)	0.0384 (17)	0.0026 (13)	0.0037 (14)	−0.0078 (13)
C19	0.0347 (17)	0.0362 (16)	0.0325 (15)	0.0051 (14)	−0.0004 (13)	0.0063 (13)
N1	0.0266 (12)	0.0263 (12)	0.0255 (11)	−0.0010 (10)	−0.0018 (10)	−0.0005 (9)
N2	0.0198 (12)	0.0276 (12)	0.0306 (13)	0.0001 (10)	0.0033 (10)	0.0010 (10)
O	0.0274 (11)	0.0255 (11)	0.0365 (12)	−0.0004 (9)	−0.0045 (9)	−0.0082 (9)

### Geometric parameters (Å, °)

**Table:**

C1—O	1.428 (3)	C13—H13	0.9500
C1—C2	1.528 (4)	C14—N1	1.471 (4)
C1—C8	1.534 (4)	C14—H14A	0.9900
C1—C14	1.549 (4)	C14—H14B	0.9900
C2—C7	1.393 (4)	C15—N1	1.462 (4)
C2—C3	1.393 (4)	C15—H15A	0.9800
C3—C4	1.388 (4)	C15—H15B	0.9800
C3—H3	0.9500	C15—H15C	0.9800
C4—C5	1.370 (5)	C16—N1	1.466 (4)
C4—H4	0.9500	C16—C17	1.513 (4)
C5—C6	1.379 (5)	C16—H16A	0.9900
C5—H5	0.9500	C16—H16B	0.9900
C6—C7	1.390 (5)	C17—N2	1.499 (4)
C6—H6	0.9500	C17—H17A	0.9900
C7—H7	0.9500	C17—H17B	0.9900
C8—C9	1.387 (4)	C18—N2	1.483 (4)
C8—C13	1.397 (4)	C18—H18A	0.9800
C9—C10	1.389 (4)	C18—H18B	0.9800
C9—H9	0.9500	C18—H18C	0.9800
C10—C11	1.380 (5)	C19—N2	1.487 (4)
C10—H10	0.9500	C19—H19A	0.9800
C11—C12	1.382 (4)	C19—H19B	0.9800
C11—H11	0.9500	C19—H19C	0.9800
C12—C13	1.383 (4)	N2—H2N	0.87 (4)
C12—H12	0.9500	O—H1O	0.69 (3)
			
O—C1—C2	111.2 (2)	C1—C14—H14A	109.2
O—C1—C8	104.8 (2)	N1—C14—H14B	109.2
C2—C1—C8	111.6 (2)	C1—C14—H14B	109.2
O—C1—C14	107.9 (2)	H14A—C14—H14B	107.9
C2—C1—C14	111.6 (2)	N1—C15—H15A	109.5
C8—C1—C14	109.5 (2)	N1—C15—H15B	109.5
C7—C2—C3	117.8 (3)	H15A—C15—H15B	109.5
C7—C2—C1	120.7 (3)	N1—C15—H15C	109.5
C3—C2—C1	121.4 (2)	H15A—C15—H15C	109.5
C4—C3—C2	121.3 (3)	H15B—C15—H15C	109.5
C4—C3—H3	119.3	N1—C16—C17	112.0 (2)
C2—C3—H3	119.3	N1—C16—H16A	109.2
C5—C4—C3	120.0 (3)	C17—C16—H16A	109.2
C5—C4—H4	120.0	N1—C16—H16B	109.2
C3—C4—H4	120.0	C17—C16—H16B	109.2
C4—C5—C6	119.7 (3)	H16A—C16—H16B	107.9
C4—C5—H5	120.2	N2—C17—C16	112.0 (2)
C6—C5—H5	120.2	N2—C17—H17A	109.2
C5—C6—C7	120.6 (3)	C16—C17—H17A	109.2
C5—C6—H6	119.7	N2—C17—H17B	109.2
C7—C6—H6	119.7	C16—C17—H17B	109.2
C2—C7—C6	120.5 (3)	H17A—C17—H17B	107.9
C2—C7—H7	119.8	N2—C18—H18A	109.5
C6—C7—H7	119.8	N2—C18—H18B	109.5
C9—C8—C13	118.0 (3)	H18A—C18—H18B	109.5
C9—C8—C1	123.3 (3)	N2—C18—H18C	109.5
C13—C8—C1	118.7 (2)	H18A—C18—H18C	109.5
C8—C9—C10	120.6 (3)	H18B—C18—H18C	109.5
C8—C9—H9	119.7	N2—C19—H19A	109.5
C10—C9—H9	119.7	N2—C19—H19B	109.5
C11—C10—C9	120.8 (3)	H19A—C19—H19B	109.5
C11—C10—H10	119.6	N2—C19—H19C	109.5
C9—C10—H10	119.6	H19A—C19—H19C	109.5
C12—C11—C10	119.2 (3)	H19B—C19—H19C	109.5
C12—C11—H11	120.4	C15—N1—C16	111.3 (2)
C10—C11—H11	120.4	C15—N1—C14	111.2 (2)
C11—C12—C13	120.1 (3)	C16—N1—C14	113.2 (2)
C11—C12—H12	119.9	C18—N2—C19	110.9 (2)
C13—C12—H12	119.9	C18—N2—C17	111.1 (2)
C12—C13—C8	121.2 (3)	C19—N2—C17	112.5 (2)
C12—C13—H13	119.4	C18—N2—H2N	110 (2)
C8—C13—H13	119.4	C19—N2—H2N	105 (2)
N1—C14—C1	111.9 (2)	C17—N2—H2N	107 (2)
N1—C14—H14A	109.2	C1—O—H1O	107 (3)

### Hydrogen-bond geometry (Å, °)

**Table:**

*D*—H···*A*	*D*—H	H···*A*	*D*···*A*	*D*—H···*A*
N2—H2N···Br	0.87 (4)	2.52 (4)	3.276 (3)	145 (4)
O—H1O···Br	0.69 (3)	2.76 (3)	3.398 (2)	156 (3)
